# CEBPG promotes acute myeloid leukemia progression by enhancing EIF4EBP1

**DOI:** 10.1186/s12935-021-02305-z

**Published:** 2021-11-07

**Authors:** You Jiang, Shui-Yan Wu, Yan-Ling Chen, Zi-Mu Zhang, Yan-Fang Tao, Yi Xie, Xin-Mei Liao, Xiao-Lu Li, Gen Li, Di Wu, Hai-Rong Wang, Ran Zuo, Hai-Bo Cao, Jing-Jing Pan, Juan-Juan Yu, Si-Qi Jia, Zheng Zhang, Xin-Ran Chu, Yong-Ping Zhang, Chen-xi Feng, Jian-Wei Wang, Shao-Yan Hu, Zhi-Heng Li, Jian Pan, Fang Fang, Jun Lu

**Affiliations:** 1 Department of Hematology, Children’s Hospital of Soochow University, No.92 Zhongnan Street, SIP, Suzhou, 215003 Jiangsu China; 2 Intensive Care Unit, Children’s Hospital of Soochow University, Suzhou, 215003 China; 3 Institute of Pediatric Research, Children’s Hospital of Soochow University, No.92 Zhongnan Street, SIP, Suzhou, 215003 China; 4 School of Basic Medicine and Biological Sciences, Soochow University, Suzhou, 215003 China

**Keywords:** CEBPG, EIF4EBP1, Acute myeloid leukemia, Proliferation, Apoptosis

## Abstract

**Background:**

Acute myeloid leukemia (AML) is a myeloid neoplasm accounts for 7.6% of hematopoietic malignancies. AML is a complex disease, and understanding its pathophysiology is contributing to the improvement in the treatment and prognosis of AML. In this study, we assessed the expression profile and molecular functions of CCAAT enhancer binding protein gamma (*CEBPG*), a gene implicated in myeloid differentiation and AML progression.

**Methods:**

shRNA mediated gene interference was used to down-regulate the expression of *CEBPG* in AML cell lines, and knockdown efficiency was detected by RT-qPCR and western blotting. The effect of knockdown on the growth of AML cell lines was evaluated by CCK-8. Western blotting was used to detect PARP cleavage, and flow cytometry were used to determine the effect of knockdown on apoptosis of AML cells. Genes and pathways affected by knockdown of *CEBPG* were identified by gene expression analysis using RNA-seq. One of the genes affected by knockdown of *CEBPG* was Eukaryotic translation initiation factor 4E binding protein 1 (*EIF4EBP1*), a known repressor of translation. Knockdown of *EIF4EBP1* was used to assess its potential role in AML progression downstream of *CEBPG*.

**Results:**

We explored the ChIP-Seq data of AML cell lines and non-AML hematopoietic cells, and found *CEBPG* was activated through its distal enhancer in AML cell lines. Using the public transcriptomic dataset, the Cancer Cell Line Encyclopedia (CCLE) and western blotting, we also found *CEBPG* was overexpressed in AML. Moreover, we observed that *CEBPG* promotes AML cell proliferation by activating *EIF4EBP1*, thus contributing to the progression of AML. These findings indicate that *CEBPG* could act as a potential therapeutic target for AML patients.

**Conclusion:**

In summary, we systematically explored the molecular characteristics of *CEBPG* in AML and identified *CEBPG* as a potential therapeutic target for AML patients. Our findings provide novel insights into the pathophysiology of AML and indicate a key role for *CEBPG* in promoting AML progression.

**Supplementary Information:**

The online version contains supplementary material available at 10.1186/s12935-021-02305-z.

## Introduction

Acute myeloid leukemia (AML) is a myeloid neoplasm that accounts for 7.6% of hematopoietic malignancies. It is caused by the oncogenic transformation of hematopoietic progenitors in the bone marrow (BM), which results in the destruction of blood tissue. AML is reported to have a long-term survival of less than 20% [1–3]. Every year there are about 18,000 new cases AML in Europe [4]. AML is a complex disease, and understanding its pathophysiology will contribute to improving the treatment and prognosis of AML [5–8].

CCAAT enhancer binding proteins (CEBPs) including CEBPA, CEBPB, CEBPD, CEBPE, CEBPG and CEBPZ, are suggested as potential biomarkers for cancer prognosis [9–14]. CEBPB plays a role in gastric cancer progression [15], and is involved in breast cancer cell migration and invasion [16]. Both CEBPB and CEBPD function in cancer cell survival [17]. CEBPD is also reported to participate in papillary thyroid carcinoma progression [18]. CEBPE is suggested as a prognostic factor for AML [19], and CEBPZ is also reported to be mutated in AML [20].

Among CEBPs, CEBPA, CEBPE and CEBPZ have been reported to function in AML development [9, 19, 20], however the role of CCAAT enhancer binding protein gamma (CEBPG) in AML is unclear. CEBPG is a member of leucine-zipper transcription factor family that plays a role in many biological processes [21–24]. Knockdown of *CEBPG* suppressed tumor growth [25]. *CEBPG* is suggested as a biomarker for lung cancer risk [26]. It is also involved in the differentiation arrest in AML [27, 28]. Although the roles of *CEBPG* in several types of cancer have been revealed, its expression profile and molecular functions in AML remain unresolved. Therefore, in this study we assess the role of *CEBPG* in AML progression.

In the present study, shRNA mediated gene interference was used to down-regulate the expression of *CEBPG* in AML cell lines, and the knockdown efficiency was detected by RT-qPCR and western blotting. The effect of *CEBPG* knockdown on the growth of AML cell lines was evaluated by Cell Counting Kit-8 (CCK-8) assays. Western blotting was used to detect poly(ADP-ribose) polymerase (PARP) cleavage, and flow cytometry was used to determine the effect of *CEBPG* knockdown on apoptosis of AML cells. Genes and pathways affected by knockdown of *CEBPG* were identified by gene expression analysis using RNA-seq.

One of the genes affected by knockdown of *CEBPG* was Eukaryotic translation initiation factor 4E binding protein 1 (*EIF4EBP1*). EIF4EBP1 is a translation repressor protein [29] that plays a role in multiple types of cancer, including lung, breast, and liver cancer [30–33]. For example, EIF4EBP1 is reported to be significantly overexpressed in hepatocellular carcinoma (HCC) tissues and is related to poor survival of patients with HCC [33]. However, the biological effect and underlying mechanism of EIF4EBP1 in AML has not been explored. Therefore, knockdown of *EIF4EBP1* was used to assess its potential role in AML progression downstream of *CEBPG*.

In the present study, we explored the ChIP-Seq data of AML cell lines and non-AML hematopoietic cells and found *CEBPG* was activated through its distal enhancer in AML cell lines. Using the public transcriptomic dataset, the Cancer Cell Line Encyclopedia (CCLE) and western blotting, we also found that *CEBPG* was overexpressed in AML. Moreover, *CEBPG* promotes AML cell proliferation by activating *EIF4EBP1*, thus contributing to the progression of AML. These findings indicate that *CEBPG* could act as a potential therapeutic target for AML patients.

## Materials and methods

### Cell lines and culture

Human AML cell lines, including NB4,THP-1, MV4-11, and K562 which was from blastic crisis of chronic myelogenous leukemia were obtained from the cell bank of the American type culture collection and cultured in RPMI medium (Termo Fisher Scientifc) containing 10% fetal bovine serum (Biological Industries, CT, USA), and 1% penicillin–streptomycin (Beyotime Biotechnology, Shanghai, China) at 37 °C in a humidified incubator with an atmosphere of 5% CO2 and tested routinely for mycoplasma.

### Lentivirus preparation and infection

Short hairpin RNA (shRNA) targeting *CEBPG* and *EIF4EBP1* (Table 1) were constructed in the pLKO.1-puro lentiviral vector (IGE BIOTECHNOLOGY LTD, Guangzhou, China). For lentivirus preparation, the envelope plasmid and packaging plasmid were purchased from Addgene (pMD2.G: #12,259; psPAX2:#12,260; Cambridge, MA, USA). pMD2.G, psPAX2 and the transfer plasmid were cotransfected into 293FT cells using polyethylenimine (linear MW 25,000 Da, 5 mg/mL, pH7.0) (cat. No. 23966–1; Polysciences, Warrington, PA, USA) according to the manufacturer’s instructions. After 6 h, the culture medium was completely replaced with fresh medium. The viral supernatant was harvested at 48 h post-transfection and filtered through a 0.22 μm filter.The leukemia cells were then infected with lentivirus in the presence of 10 μg/mL Polybrene (Sigma–Aldrich) for 24 h. Stable cell lines were selected with puromycin (Sigma-Aldrich).

**Table 1:** shRNAs used to knockdown *CEBPG* and *EIF4EBP1*

Name	Sequence
Homo-CEBPG -sh1	CCGGGATTTGTTTCTTGAGCATGCACTCGAG
	TGCATGCTCAAGAAACAAATCTTTTTGAATT
Homo-CEBPG -sh2	CCGGTGGCGACAATGCAGGACAGTACTCGA
	GTACTGTCCTGCATTGTCGCCATTTTTGAATT
Homo-CEBPG -sh3	CCGGGCAACGCCGAGAGAGGAACAACTCGA
	GTTGTTCCTCTCTCGGCGTTGCTTTTTGAATT
Homo-EIF4EBP1-sh1	CCGGGCCAGAGCCACCTGCGCAATACTCGA
	GTATTGCGCAGGTGGCTCTGGCTTTTTGAATT
Homo-EIF4EBP1-sh2	CCGGGCAATAGCCCAGAAGATAAGCCTCGA
	GGCTTATCTTCTGGGCTATTGCTTTTTGAATT
Homo-EIF4EBP1-sh3	CCGGGCGGTGAAGAGTCACAGTTTGCTCGA
	GCAAACTGTGACTCTTCACCGCTTTTTGAATT

### Cell viability assay

Leukemia cells were seeded in 96-well plates at a density of 1 × 10^3^ cells per well. The cell viability was determined by Cell Counting kit-8 (CCK8) assay (Dojindo Molecular Technologies, Tokyo, Japan) according to the manufacturer’s instructions. Cell proliferation was calculated as a percentage of that in cells in control medium. Each concentration was tested in triplicate and repeated in at least three independent experiments. The calculation was performed by Graph Prism software 7.0 (GraphPad Software Inc., San Diego, CA, USA).

### RNA preparation and real-time PCR expression analysis

Total RNA was extracted from cell pellets using TRIzol®reagent (Invitrogen, CA, USA), according to the manufacturer’s protocol. For cDNA synthesis, 1 µg of total RNA was converted to cDNA using a High-Capacity cDNA Reverse Transcription Kit (Applied Biosystems, CA, USA). Quantitative real-time PCR analysis was carried out using LightCycler® 480 SYBR Green I Master mix (cat. No. 04707516001; Roche, Penzberg, Germany) with a LightCycler 480 Real Time System (Roche), according to the manufacturer’s protocol. mRNA expression levels were calculated using the Ct method with glyceraldehyde 3-phosphate dehydrogenase (GAPDH) expression as an internal reference. Primer sequences are listed in Table 2.

**Table 2:** Primers used for qRT-PCR analyses

Name	Sequence (5’- > 3’)
CEBPG Forward	GAAAAAGAGCCGGTTGAAAAGC
CEBPG Reverse	ACTGTACGTTGTCTGCAAGGT
EIF4EBP1 Forward	CTATGACCGGAAATTCCTGATGG
EIF4EBP1 Reverse	CCCGCTTATCTTCTGGGCTA
GAPDH Forward	TGCACCACCAACTGCTTAG
GAPDH Reverse	GATGCAGGGATGATGTTC
PDGFB Forward	CTCGATCCGCTCCTTTGATGA
PDGFB Reverse	CGTTGGTGCGGTCTATGAG
SRC Forward	TGGCAAGATCACCAGACGG
SRC Reverse	GGCACCTTTCGTGGTCTCAC
PLCG1 Forward	GGAAGACCTCACGGGACTTTG
PLCG1 Reverse	GCGTTTTCAGGCGAAATTCCA
EIF4E Forward	ATGTGGCGCTGTTGTTAATGT
EIF4E Reverse	CTGCGTGGGACTGATAACCAA
AXL Forward	GTGGGCAACCCAGGGAATATC
AXL Reverse	GTACTGTCCCGTGTCGGAAAG
PIK3R2 Forward	TCACCTTCTGCTCCGTTGTG
PIK3R2 Reverse	GGAGGTCCGTGTGTACTCTTC
MET Forward	AGCGTCAACAGAGGGACCT
MET Reverse	GCAGTGAACCTCCGACTGTATG

### Western blotting analysis

Western blotting analysis was conducted using the following primary antibodies: CEBPG (cat. sc-517003; 1:500; Santa Cruz Biotechnology, Inc. Dallas, Texas,USA), EIF4EBP1 (cat. #9644,1:1000; Cell Signaling Technology, Boston, MA, USA), and PARP (cat. No. 9542; 1:1000; Cell Signaling Technology), with glyceraldehyde 3-phosphate dehydrogenase (GAPDH) (cat. No. MA3374; 1:1000; Millipore) as a reference protein. Peroxidase-conjugated Afniure goat anti-rabbit IgG (H + L) (cat.111-035-003; 1:5000) and goat anti-mouse IgG (H + L) (cat. No. 115-035-003; 1:5000) secondary antibodies were purchased from Jackson ImmunoResearch Laboratories, Inc. (West Grove, PA, USA). ImageJ software was used for band quantifcation. Then, protein levels were determined using a GAPDH antibody for normalization.

### Cell apoptosis assay

Leukemia cells (MV4-11, NB4, and K562 cell lines) were infected with lentivirus in the presence of 10 μg/mL Polybrene (Sigma-Aldrich) for 24 h. Stable cell lines were selected with puromycin (Sigma-Aldrich). Following 4 days incubation, leukemia cells were harvested and washed with cold PBS, suspended in 1 × binding bufer, and stained with fuorescein isothiocyanate (FITC)-Annexin V antibody and PI solution using an FITC-Annexin V apoptosis kit (cat. No.556420; BD Biosciences, Franklin Lakes, NJ, USA), according to the manufacturer’s instructions. Cell apoptosis was analyzed by flow cytometry (Beckman Gallios™ Flow Cytometer; Beckman).

### RNA-seq and data processing

RNA-seq was carried out according to the protocols suggested by Novogene, Beijing, China. First, total RNA was reverse transcribed to cDNA for library construction, and the cDNA library was then sequenced. The raw reads were filtered and clean reads were mapped according to HISAT. The gene expression level (as fragments per kilobase of exon model per million reads mapped) was then calculated. Differentially expressed genes (P < 0.05 and fold-change > 2 or fold-change < 0.5) were identified using DESeq2 analysis. For enrichment analysis, differentially expressed genes were analyzed using the DAVID Bioinformatics Resources v6.8 online server (https://david.ncifcrf.gov).

### Chromatin immunoprecipitation (ChIP)

3–5 × 10^7^ cells were crosslinked with 1% formaldehyde for 10 min and neutralized with 1.25 M glycine for 5 min at room temperature. Fixed cells were harvested, lysed, and sonicated using a Bioruptor (Diagenode, Liège, Belgium). Sonicated chromatin was incubated with anti-histone H3 (acetyl K27) antibody (cat. No. ab4729; Abcam, Cambridge, UK) overnight at 4 °C. DNA was eluted and purified using a QIAquick PCR purification kit (cat. No. 208106; Qiagen, Hilden, Germany). Samples were sequenced on a novaseq 6000 platform (Novogene Bioinformatics Technology Co., Ltd. Beijing, China). Raw data of ChIP-Seq H3K27ac analysis for NB4 cell line was aligned to the reference genome (UCSC hg38) using Bowtie2 (v 2.3.5) [34], with alignment parameters -p 4 -q -x. Peaks were identified using MACS2 (v2.0.9) [35], with parameters -g hs -n test -B -q 0.01. The bedgraph files generated by MACS2 were converted to bigwig files using the UCSC bedGraphToBigWig tool, and then bigwig files were visualized by Integrative Genomics Viewer (IGV) [36].

### Public ChIP-Seq data collection and analysis

In this study, we searched public ChIP-Seq H3K27ac datasets of AML cell lines and non-AML hematopoietic cells in the Cistrome database (http://www.cistrome.org/). The ChIP-Seq datasets of H3K27ac and CEBPG in K562 cell line were also obtained in the Cistrome database. The bigwig files of those datasets obtained (GSE113040, GSE80779, GSE76783, GSE79899, GSE71809, GSE107147, GSE70660, GSE93372, GSE105532, GSE70482) were further visualized by Integrative Genomics Viewer (IGV) [36].

### Statistical analysis

The association between *EIF4EBP1* expression and overall survival of AML patients were assessed using the Kaplan–Meier analysis. Comparison between two groups was carried out using the Student’s t-test or the Mann–Whitney u test. Statistical analysis was carried out by GraphPad Prism 7.0 (GraphPad Software, Inc., La Jolla, CA, USA). Statistically significant P values are indicated as *P < 0.05, ** P < 0.01, ***P < 0.001, and ****P < 0.0001.

## Results

### *CEBPG* is activated through its distal enhancer and is overexpressed in AML cell lines

**Fig. 1:**
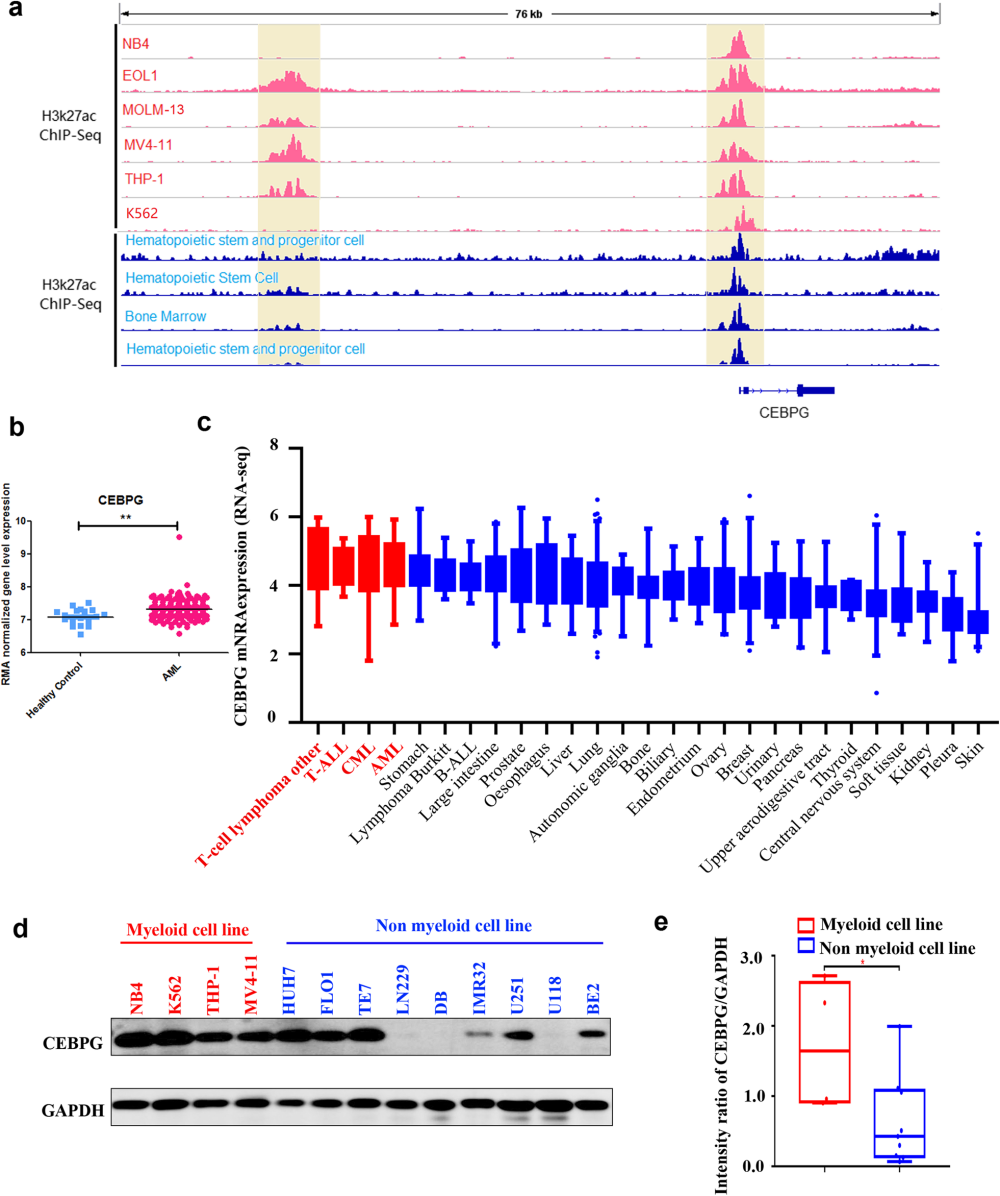
**a** ChIP-Seq data analysis results for *CEBPG* of AML cell lines (K562 cell line included, tracks 1–6) and non-AML hematopoietic cells (tracks 7–10); **b** expression pattern of *CEBPG* between AML patients and healthy controls in public transcriptomic dataset (GSE114868); **c** *CEBPG* was highly expressed in hematologic tumors including AML according to the Cancer Cell Line Encyclopedia (CCLE; https://portals.broadinstitute.org/ccle); **d** western blotting results of the expression levels of CEBPG in AML/non-AML cell lines; **e** CEBPG markedly upregulated in AML cell lines compared with non-AML cell lines by western blotting

By interrogating ChIP-Seq data of AML cell lines (Fig, 1a, tracks 1–6, K562 cell line also included) and non-AML hematopoietic cells (Fig. 1a, tracks 7–10), we found that the enhancer region of *CEBPG* in AML cell lines showed coincident H3K27ac signals that were not present in non-AML hematopoietic cells, suggesting a potential role in transcription regulation. Then, we assessed the expression pattern of *CEBPG* between AML patients and healthy controls in a public transcriptomic dataset (GSE114868) [37], and found that *CEBPG* was more highly expressed in AML samples (Fig. 1b) relative to that in healthy control samples (the differentially expressed genes between AML and control samples in dataset GSE114868 are listed in Additional file 1: Table S1). Moreover, the Cancer Cell Line Encyclopedia (CCLE; https://portals.broadinstitute.org/ccle) which includes *CEBPG* mRNA expression profiles for multiple cancer cell lines, showed that CEBPG was highly expressed in hematologic malignancies including AML (Fig. 1c). We also assessed the levels of CEBPG in AML and non-AML cell lines using western blotting, and found higher levels of CEBPG in AML cell lines than in non-AML cell lines (Fig. 1d and e). Collectively, these data suggested that *CEBPG* is activated through its distal enhancer and overexpressed in AML.

### CEBPG is oncogenic and promotes AML cell proliferation

**Fig. 2:**
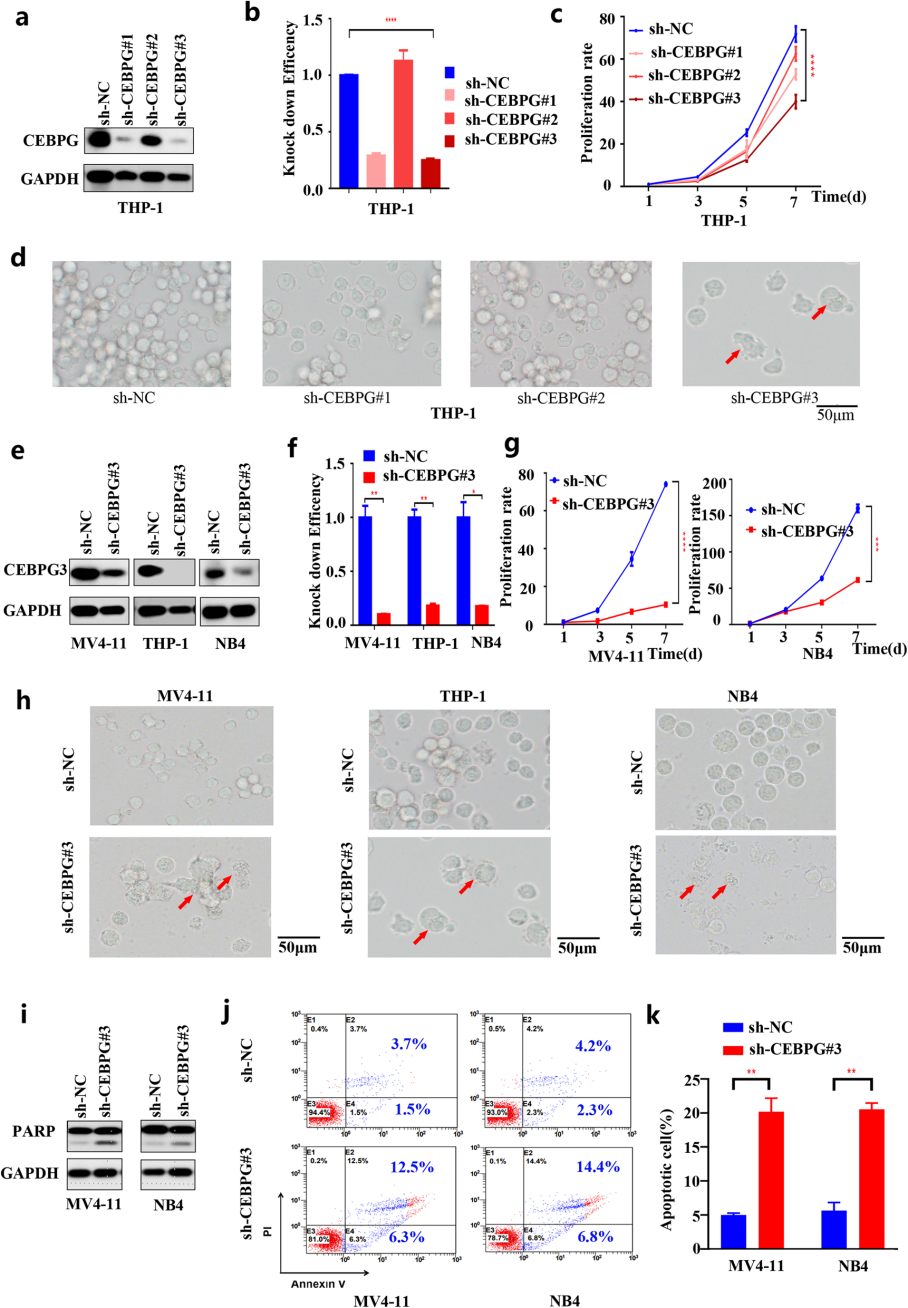
**a** Knockdown efficiency of *CEBPG* was evaluated in THP-1 cell line by western blotting. **b** Knockdown efficiency of *CEBPG* was evaluated in THP-1 cell line by qPCR. **c** Knockdown of *CEBPG* significantly inhibited the proliferation rates of THP-1 cell line. **d** Knockdown of *CEBPG* significantly inhibited the proliferation rates of THP-1 cell line. **e** Knockdown efficiency of *CEBPG* was evaluated in MV4-11, THP-1, and NB4 cell lines by western blotting. **f** Knockdown efficiency of *CEBPG* was evaluated in MV4-11, THP-1, and NB4 cell lines by qPCR. **g** Knockdown of *CEBPG* significantly inhibited the proliferation rates of MV4-11 and NB4 cell lines. **h** Knockdown of *CEBPG* significantly inhibited the proliferation rates of MV4-11, THP-1, and NB4 cell lines. **i** PARP was increased in both MV4-11 and NB4 cell lines upon knockdown of *CEBPG*. **j** Flow cytometry showed that knockdown of *CEBPG* increased the apoptotic rates of MV4-11 and NB4 cell lines. **k** Knockdown of *CEBPG* increased the apoptotic rates of MV4-11 and NB4 cell lines

To address the biological significance of *CEBPG*, we selected three AML cell lines with high CEBPG protein levels shown in Fig. 1d (THP-1, NB4 and MV4-11) and performed shRNA-mediated knockdown of *CEBPG* using three independent shRNAs (Table 1). Knockdown efficiency of *CEBPG* was evaluated using western blotting and qPCR (Fig. 2a, b, e and f). Notably, knockdown of *CEBPG* significantly inhibited the proliferation rates of all 3 AML cell lines (Fig. 2c, d, g and h). We also assessed the level of the apoptotic protein PARP using western blotting and found that PARP levels were increased in both MV4-11 and NB4 cell lines upon knockdown of *CEBPG* (Fig. 2i). Knockdown of *CEBPG* also increased the apoptotic rates of MV4-11 and NB4 cell lines (Fig. 2j and k). Altogether, these data suggested that *CEBPG* is oncogenic and contributes to the proliferation of AML cells.

### *CEBPG* activates *EIF4EBP1* in AML cell lines

**Fig. 3:**
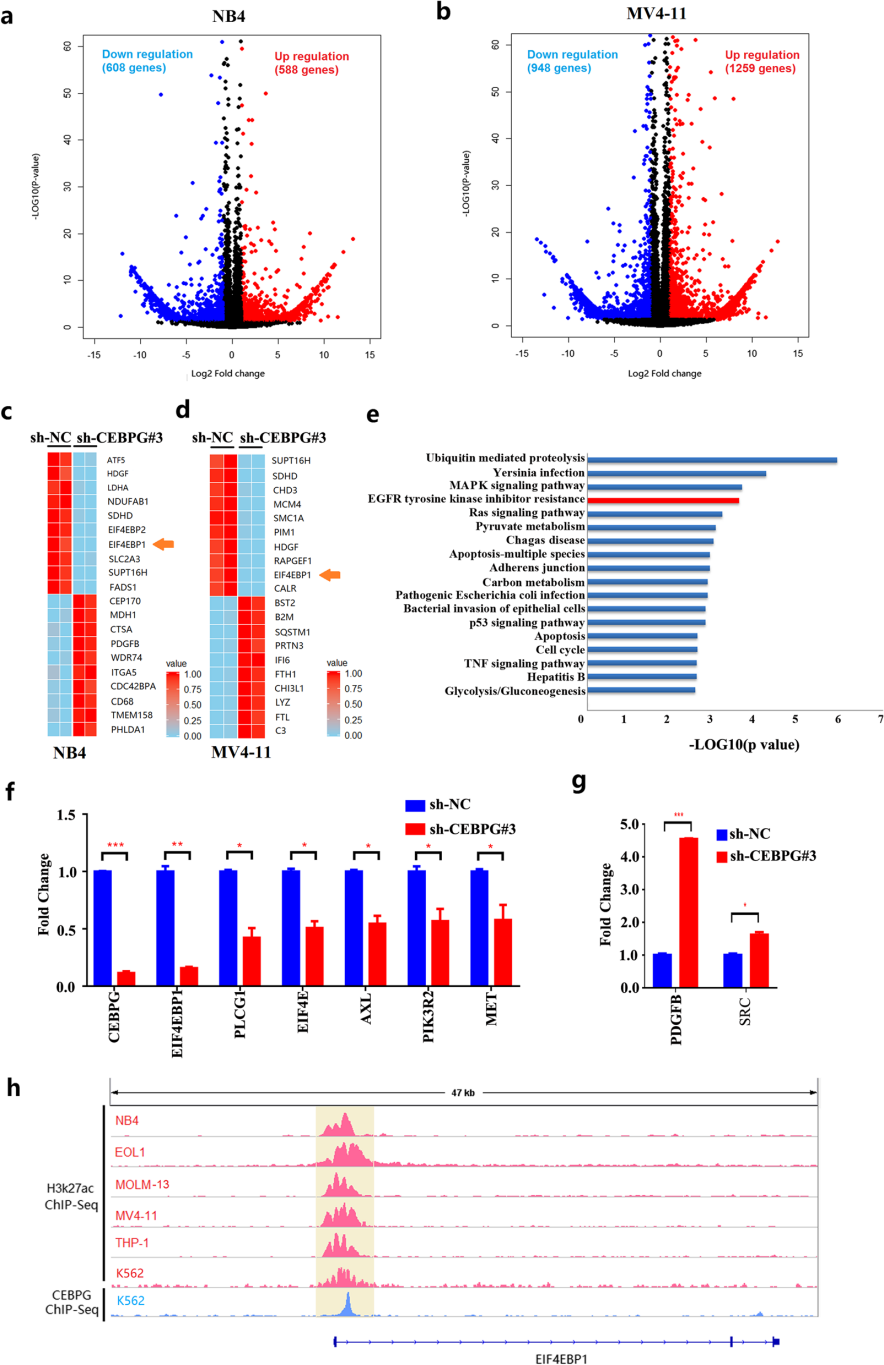
**a** Volcano Plot of RNA-seq results for NB4 cell line in either the absence or presence of CEBPG. **b** Volcano Plot of RNA-seq results for MV4-11 cell line in either the absence or presence of CEBPG. **c** Top 10 downregulated and top 10 upregulated genes upon *CEBPG* knockdown in NB4 cell line. **d** Top 10 downregulated and top 10 upregulated genes upon *CEBPG* knockdown in MV4-11 cell line. **e** Enrichment analysis results of all DEGs by using the KEGG Pathway Database. **f** qRT-PCR results of 6 genes (*EIF4EBP1*, *PLCG1*, *EIF4E*, *AXL*, *PIK3R2* and *MET*) from EGFR tyrosine kinase inhibitor resistance signaling pathway in NB4 cell line when silencing *CEBPG*. **g** qRT-PCR results of 2 genes (*PDGFB* and *SRC*) from EGFR tyrosine kinase inhibitor resistance signaling pathway in NB4 cell line when silencing *CEBPG*. **h** ChIP-Seq data of AML cell lines and K562 cell line showed that the promoter region of *EIF4EBP1* had coincident H3K27ac signals (tracks 1–6), ChIP-Seq data of K562 cell line further indicated that *EIF4EBP1* was bound by CEBPG at its TSS-proximal regions (track 7)

To reveal potential targets responsible for CEBPG-promoted AML cell proliferation, RNA-seq analyses were performed on NB4 and MV4-11 cell lines comparing shRNA control cells with *CEBPG* knockdown cells. A total of 1196 and 2207 differently expressed genes (DEGs) were identified upon *CEBPG* knockdown, in NB4 and MV4-11 cell lines respectively (Log2 |fold change|> 1, P < 0.05, Fig. 3a and b). *EIF4EBP1* was included in the top 10 downregulated genes upon *CEBPG* knockdown in both NB4 and MV4-11 cell lines (Fig. 3c and d). Next, we conducted a functional enrichment analysis of all DEGs using the KEGG Pathway Database. The results showed a significant enrichment for EGFR tyrosine kinase inhibitor resistance signaling (ranking 4^th^), which involves *EIF4EBP1* (Fig. 3e). Therefore, *EIF4EBP1* was selected for in-depth investigation. To further determine the regulation of *CEBPG* on EGFR tyrosine kinase inhibitor resistance signaling and *EIF4EBP1*, a total of 8 genes (*EIF4EBP1*, *PLCG1*, *EIF4E*, *AXL*, *PIK3R2*, *MET*, *PDGFB* and *SRC*) from the EGFR tyrosine kinase inhibitor resistance signaling pathway was selected for qRT-PCR validation. In accordance with the RNA-Seq results, the mRNA levels of 6 of these genes, including *EIF4EBP1*, were downregulated while 2 genes were upregulated in NB4 cells in response to *CEBPG* silencing (Fig. 3f and g). Additionally, ChIP-Seq data of AML cell lines and K562 cell line showed that the promoter region of *EIF4EBP1* had coincident H3K27ac signals (Fig. 3h, tracks 1–6), while the ChIP-Seq data from K562 cells further indicated that *EIF4EBP1* was bound by CEBPG at its TSS-proximal regions (Fig. 3h, track 7), suggesting a potential role for CEBPG in the transcriptional regulation of *EIF4EBP1*. Therefore, we next investigated the role of *EIF4EBP1* in NB4 and K562 cells.

### *EIF4EBP1* knockdown interferes with AML cell proliferation and increases apoptosis

**Fig. 4:**
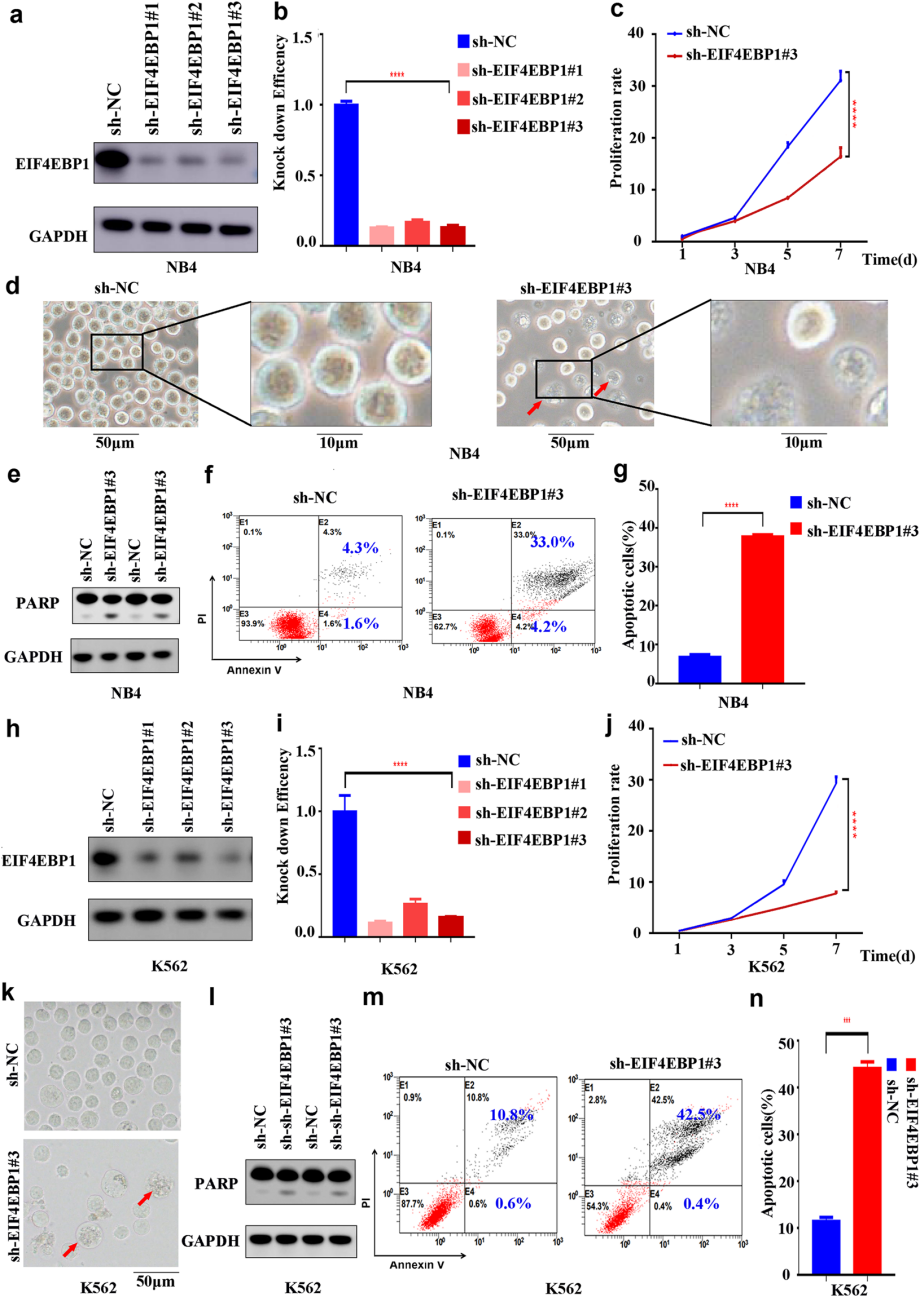
**a** Knockdown efficiency of *EIF4EBP1* was evaluated in NB4 cell line by western blotting. **b** Knockdown efficiency of *EIF4EBP1* was evaluated in NB4 cell line by qPCR. **c** Knockdown of *EIF4EBP1* significantly inhibited the proliferation rates of NB4 cell line. **d** Knockdown of *EIF4EBP1* significantly inhibited the proliferation rates of NB4 cell line. **e** PARP was increased in NB4 cell line upon knockdown of *EIF4EBP1*. **f** Flow cytometry showed that knockdown of *EIF4EBP1* increased the apoptotic rates of NB4 cell line. **g** Knockdown of *EIF4EBP1* increased the apoptotic rates of NB4 cell line. **h** Knockdown efficiency of *EIF4EBP1* was evaluated in K562 cell line by western blotting. **i** Knockdown efficiency of *EIF4EBP1* was evaluated in K562 cell line by qPCR. **j** Knockdown of *EIF4EBP1* significantly inhibited the proliferation rates of K562 cell line. **k** Knockdown of *EIF4EBP1* significantly inhibited the proliferation rates of K562 cell line.** l** PARP was increased in K562 cell line upon knockdown of *EIF4EBP1*. **m** Flow cytometry showed that knockdown of *EIF4EBP1* increased the apoptotic rates of K562 cell line. **n** Knockdown of *EIF4EBP1* increased the apoptotic rates of K562 cell line

To evaluate the biological significance of *EIF4EBP1*, we selected 2 cell lines (NB4 and K562) and knocked down *EIF4EBP1* in both cell lines using three independent shRNAs (Table 1). Knockdown efficiency of *EIF4EBP1* was evaluated using western blotting and qPCR (Fig. 4a, b, h and i). Notably, knockdown of *EIF4EBP1* significantly inhibited the proliferation rates of both cell lines (Fig. 4c, d, j and k). We also assessed the expression level of the apoptotic protein PARP using western blotting and found that PARP levels increased in both NB4 and K562 cell lines upon knockdown of *EIF4EBP1* (Fig. 4e and l). Knockdown of *EIF4EBP1* also increased the apoptotic rates of NB4 and K562 cell lines (Fig. 2f, g, 4m and n). Collectively, these data suggested that EIF4EBP1 is required to sustain proliferation and survival of AML cells.

### Identification of EIF4EBP1 as an unfavorable prognostic factor for AML patients

**Fig. 5:**
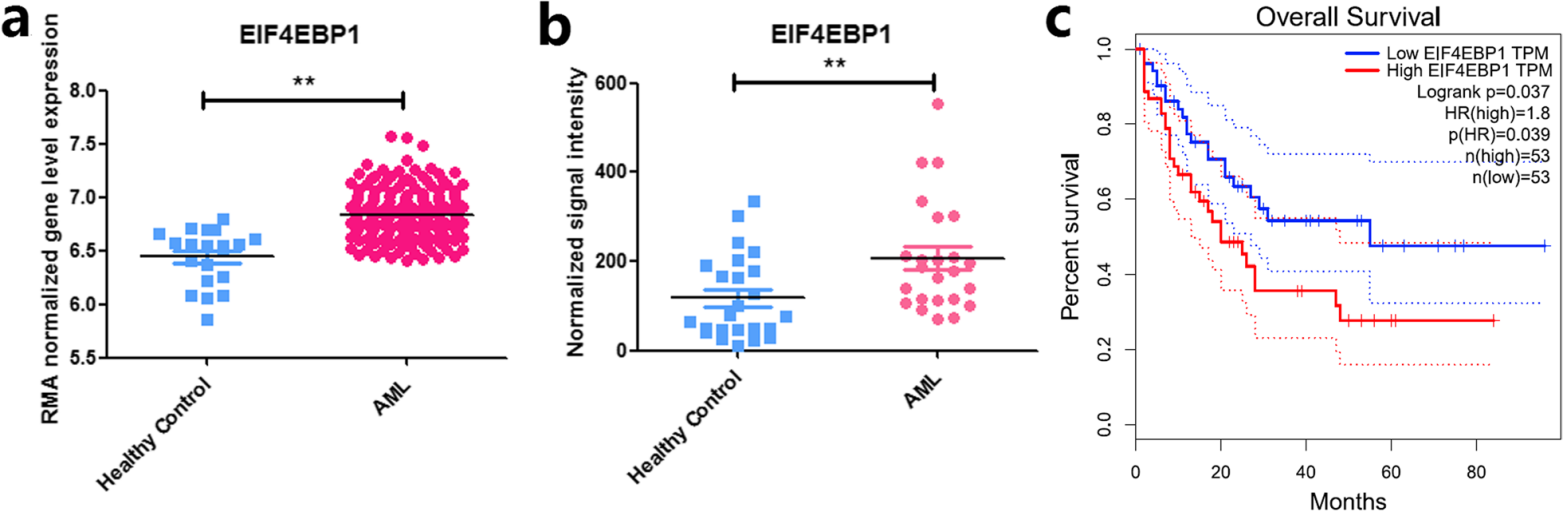
**a** *EIF4EBP1* was significantly overexpressed in AML samples in public transcriptomic dataset GSE114868. **b** *EIF4EBP1* was significantly overexpressed in AML samples in public transcriptomic dataset GSE142700. **c** Base on the online tool http://gepia.cancer-pku.cn/, the overall survival of AML patients with higher *EIF4EBP1* expression was significantly poorer than those with lower *EIF4EBP1* expression

We assessed the expression pattern of *EIF4EBP1* between AML patients and healthy controls in two public transcriptomic datasets (GSE114868 and GSE142700) [37]. The results showed that *EIF4EBP1* was significantly overexpressed in AML samples in both datasets (Fig. 5a and b). To further explore the prognostic value of *EIF4EBP1*, we used the online tool http://gepia.cancer-pku.cn/ and the result showed that the overall survival of AML patients with higher *EIF4EBP1* expression was significantly poorer than those with lower *EIF4EBP1* expression (Fig. 5c). These results suggested that EIF4EBP1 represents a negative prognostic factor for AML patients.

## Discussion

AML is an aggressive malignancy with poor prognosis [8]. It is a complex disease and a detailed understanding of its pathophysiology is required to improve the treatment and prognosis of AML [5–8].

CCAAT enhancer binding proteins (CEBPs) including CEBPA, CEBPB, CEBPD, CEBPE, CEBPG and CEBPZ, are suggested as potential biomarkers for cancer prognosis [9–14]. Among CEBPs, CCAAT enhancer binding protein gamma (CEBPG), a member of leucine-zipper transcription factor family, has been implicated in multiple cancers [25–28]. For example, it is reported that CEBPG significantly promotes the proliferation and migration of esophageal squamous cell carcinoma (ESCC) cells, and is thus suggested as a prognostic factor for patients with ESCC [21].

Although a role for CEBPG in myeloid differentiation has been demonstrated [27, 28], if and how it contributes to the pathogenesis of AML is unclear. Here, we explored the function of CEBPG in AML and found that CEBPG is upregulated in AML and contributes to the proliferation of AML cells. We also demonstrated that CEBPG promotes AML cell proliferation by activating *EIF4EBP1* in AML cell lines.

Eukaryotic translation initiation factor 4E binding protein 1 (*EIF4EBP1*) gene encodes a translation repressor protein [29]. This protein plays a role in multiple cancer types, including lung, breast, and liver cancer [30–33]. For example, EIF4EBP1 is reported to be significantly overexpressed in hepatocellular carcinoma (HCC) tissues and is related to poor survival of HCC patients [33]. However, the biological effect and underlying mechanism of EIF4EBP1 in AML has not been explored. In this study, we found the knockdown of *EIF4EBP1* significantly inhibited proliferation and increases apoptosis in NB4 and K562 cells. Furthermore, in two public transcriptomic datasets (GSE114868 and GSE142700) [37], *EIF4EBP1* was observed to be significantly overexpressed in AML samples. EIF4EBP1 was also identified as an unfavorable prognostic factor for AML patients using the online tool http://gepia.cancer-pku.cn/. Taken together, these results suggested that EIF4EBP1 is involved in the pathogenesis of AML and represents a negative prognostic factor for AML patients.

In summary, we explored the function of CEBPG in AML and identified CEBPG as a potential therapeutic target for AML patients. Our findings provide novel insights into the pathophysiology of AML and elucidated a crucial role of CEBPG in promoting AML progression.

## Supplementary Information

**Additional file 1: Table S1**. Differentially expressed genes between AML and control samples in dataset GSE114868.

## Data Availability

The data used and/or analyzed during the current study are available from the corresponding author on reasonable request (GSE178287).

## Abbreviations

AML: Acute myeloid leukemia
ChIP: Chromatin immunoprecipitation
CEBPG: CCAAT enhancer binding protein gamma
EIF4EBP1: Eukaryotic translation initiation factor 4E binding protein 1
